# A novel adaptation facilitates seed establishment under marine turbulent flows

**DOI:** 10.1038/s41598-019-56202-7

**Published:** 2019-12-23

**Authors:** Gary A. Kendrick, Andrew W. Pomeroy, Robert J. Orth, Marion L. Cambridge, Jeremy Shaw, Lukasz Kotula, Ryan J. Lowe

**Affiliations:** 1 The University of Western Australia, School of Biological Sciences, 35 Stirling Hwy, 6009 Crawley, W.A. Australia; 2 The University of Western Australia, Oceans Institute, Crawley, Australia; 3 The University of Western Australia, Oceans Graduate School, Crawley, Australia; 4 Virginia Institute of Marine Science, College of William & Mary, 1375 Greate Rd., Gloucester Pt., VA, 23062 USA; 5 The University of Western Australia, Centre for Microscopy Characterisation and Analysis, Crawley, Australia; 6 The University of Western Australia, UWA School of Agriculture and Environment, Crawley, Australia

**Keywords:** Biooceanography, Evolutionary ecology, Population dynamics, Marine biology, Physical oceanography

## Abstract

Seeds of Australian species of the seagrass genus *Posidonia* are covered by a membranous wing that we hypothesize plays a fundamental role in seed establishment in sandy, wave swept marine environments. Dimensions of the seed and membrane were quantified under electron microscopy and micro-CT scans, and used to model rotational, drag and lift forces. Seeds maintain contact with the seabed in the presence of strong turbulence: the larger the wing, the more stable the seed. Wing surface area increases from *P. sinuosa* < *P. australis* < *P.coriacea* correlating with their ability to establish in increasingly energetic environments. This unique seed trait in a marine angiosperm corresponds to adaptive pressures imposed on seagrass species along 7,500 km of Australia’s coastline, from open, high energy coasts to calmer environments in bays and estuaries.

## Introduction

Hooks, hairs, wings and awls on plant seeds have been a constant source of novelty and research focus in terrestrial dispersal biology^1,2^. Many of these structures enhance wind dispersal of seeds. Seeds in marine environments are water dispersed (via waves, tides, currents) where mean flow is usually one or two orders of magnitude weaker than the mean atmospheric wind velocity (0.1–1 m s^−1^ versus 1–10 m s^−1^). However, forces imposed on seeds are approximately three orders of magnitude greater than those exerted on the same seeds when exposed to the same velocities in air, as seawater is approximately 1000 times denser than air.

Despite these large forces, seagrass seeds generally do not disperse far from their parent plant and are often released within or near the sediment surface^3^. If seeds do disperse over large distances (10s-100s kms) they are usually encased within floating fruit (pericarps) or plant parts (rhipidia or spathes)^3,4^ and when released the negatively buoyant seed quickly settles to the seabed^3^. The seagrass genus *Posidonia* has floating fruits and some species have a prominent membranous wing on their seeds, similar to that observed in winged seeds of some terrestrial plants^1^. Yet ecological drivers and evolutionary pressures on the formation of a membranous wing in the seeds of *Posidonia* species do not link directly to any significant increase in dispersal distances, which are dominated by the transportation of floating fruit^5,6^. This contrasts to the evolution of morphologically similar wings in terrestrial families of plants^1,7^.

We hypothesize that this membranous wing has instead evolved to reduce hydrodynamic forces on the seeds on the seabed, and hence stabilize their orientation and position, until roots grow and firmly anchor the developing seedling. This helps newly settled seeds overcome their greatest threat to establishment: the hydrodynamics of the marine environment.

## Results

**Figure 1:**
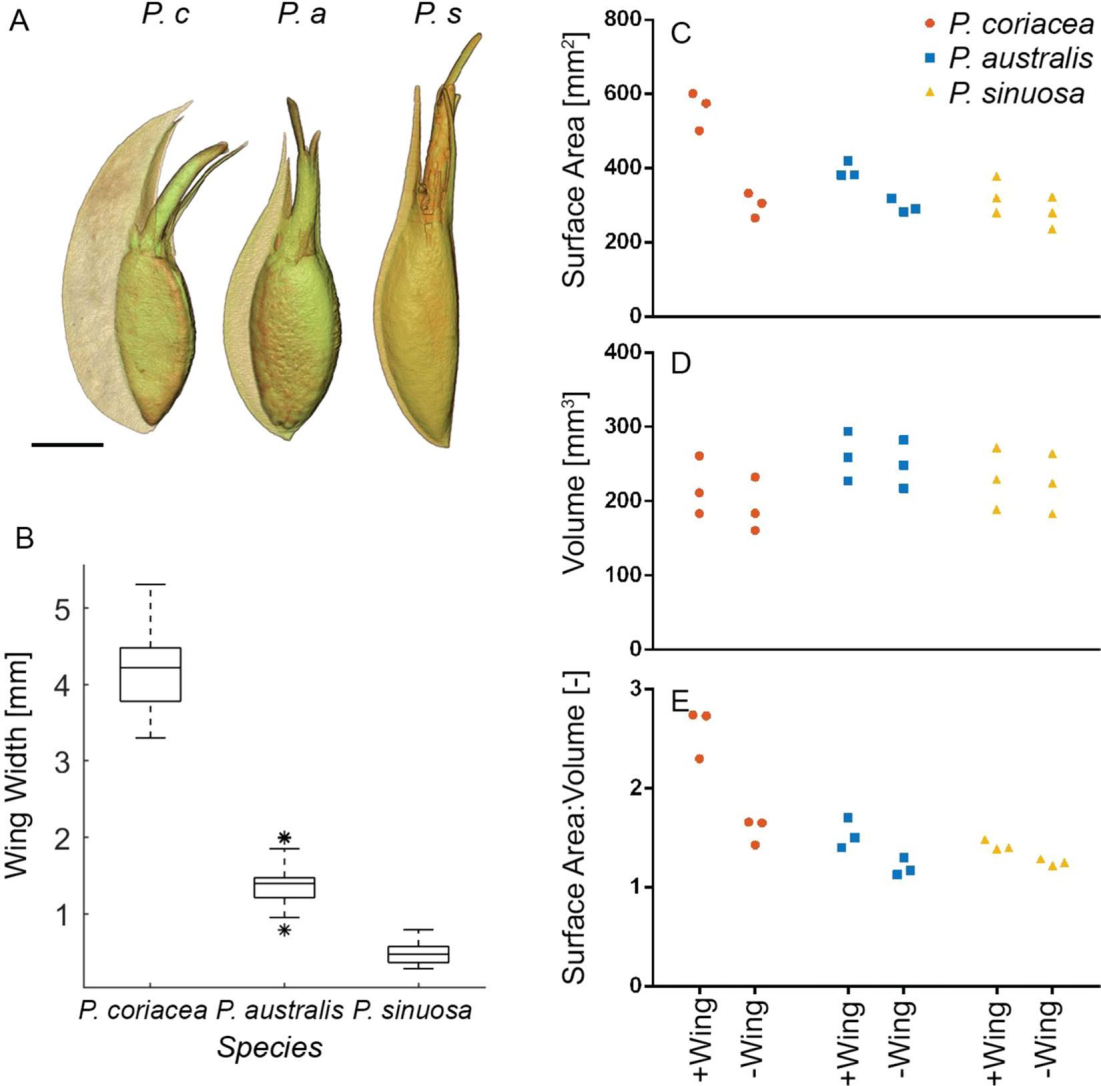
(**A**) Micro-CT scans *Posidona coriacea (P. c), P. australis (P. a)* and *P. sinuosa (P. s)* showing size of wing in relation to the seed (scale = 5000 µm), (**B**) differences in the width of the wing taken at the widest point for seeds (n = 30) for *Posidonia* species, (**C**) surface Area, (**D**) volume and (**E**) surface Area to Volume ratio determined from micro-CT are shown for 3 paired replicate seeds for each species with and without the wing.

**Figure 2:**
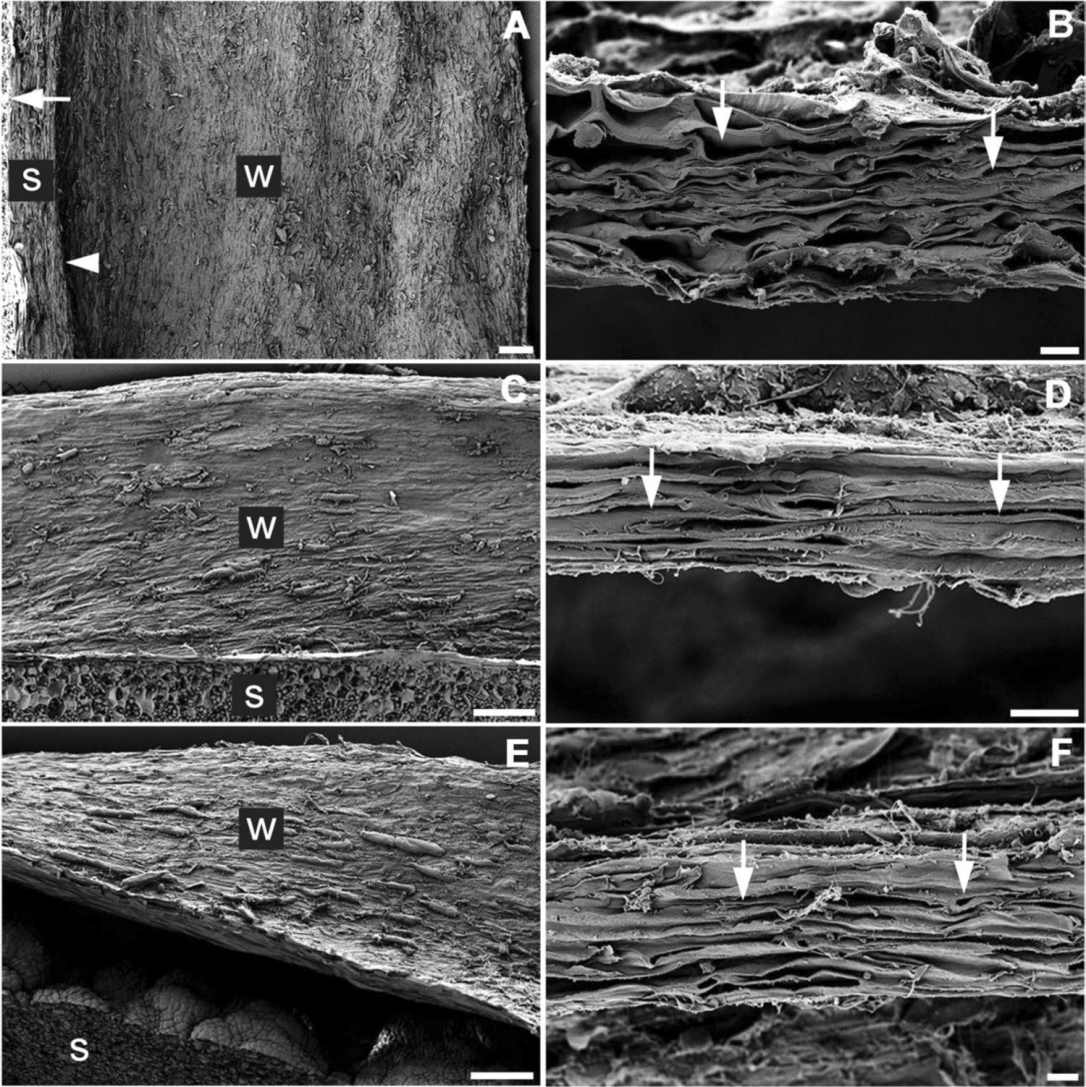
Scanning electron micrographs of surface (**A**,**C**,**E**) and cross-section (**B**,**D**,**F**) of a keel of *P. coriacea* (**A**,**B**), *P. australis* (**C**,**D**) *and P. sinuosa* (**E**,**F**). Bars = 200 µm (**A**,**C**,**E**) or 20 µm (**B**,**D**,**F**). The arrowhead in (**A**,**C**,**E**) indicates base of the membranous wing; W, wing; S, seed. The arrows in (**B**,**D**,**F**) indicate ‘flattened’ cells.

To test our hypothesis, X-ray micro-computed Tomography (X-ray micro-CT) scans were used to digitally dissect seeds for three species of *Posidonia* (*P. coriacea*, *P.australis* and *P. sinuosa*, Fig. 1A, see Supplementary Movie S1, Tables S1 and S2) to precisely determine the composition, size, surface area and shape of the membranous wing. Scanning Electron Microscopy (SEM) observations revealed that the wing of all three species is unsculptured, smooth with wavy surfaces (Fig. 2) and consists of 3–5 layers of irregular elongated, thickened and flattened cells developed from the ovary wall (Fig. 2). There is no differentiation in the structure of the winged membrane covering seeds between the three *Posidonia* species, other than marked differences in the wing width (Fig. 1A,B), which is also related to the different hydrodynamic regimes where they typically colonize (Table 1). Mean maximum width of the wing (Fig. 1B) of 30 seeds was significantly different between the three *Posidonia* species (ANOVA (ln transformed), F_2,87_ = 753.7, p < 0.001). Surface area (Fig. 1C), surface area to volume ratios (Fig. 1E) (p < 0.01) and volume (Fig. 1D) (p < 0.05) were significantly smaller (n = 3, one-tailed paired t-tests, Table 2) when the membranous wing was removed from seeds suggesting the role of the wing is to increase seed surface area relative to volume.

**Table 1:** The swell and wind wave climate where seeds of *Posidonia coriacea*, *P. sinuosa* and *P. australis* settle compared to the congeneric *P. oceanica* in the Mediterranean.

	Region	Minimum Depth of Meadows	Ocean Swells	Wind waves	
				height	season
*P. coriacea*	SW Australia	Exposed coasts, deeper inner shelf regions (>3 m)	2–3 m mean 8–12 m max^37,38^	1.5 m	summer
*P. australis*	S Australia	Estuaries, sheltered coasts	1.5 m mean 7 m max^38^	0.5 m	summer
*P. sinuosa*	S Australia	Sheltered coasts, deeper coastal regions (>1 m)	1.5 m mean 7 m max^38^	0.5 m	summer
*P. oceanica*	W. Mediterranean	Sheltered coasts, deep inner shelf regions (>1 m)	<0.5–2 m^39,40^	<0.25–0.5 m	summer

**Table 2:** One tailed paired t tests of surface area (SA), volume (V) and surface area to volume ratios (SA/V) between paired seeds with the membranous keel attached and when removed for *P. coriacea*, *P. australis* and *P. sinuosa*.

Species	variable	d.f.	t-test	p-value	95% CI	Mean difference
*P. coriacea*	Surface Area (SA)	2	19.316	0.0013	309.399	364.5
	Volume (V)	2	15.636	0.0020	41.747	51.333
	SA/V ratio	2	11.942	0.0035	1.055	1.397
*P. australis*	Surface Area (SA)	2	27.398	0.0007	162.645	183.167
	Volume (V)	2	6.371	0.0119	21.307	39.333
	SA/V ratio	2	10.392	0.0046	0.431	0.600
*P. sinuosa*	Surface Area (SA)	2	8.832	0.0063	86.465	129.167
	Volume (V)	2	12.333	0.0326	28.240	37.000
	SA/V ratio	2	20.057	0.0012	0.347	0.407

**Figure 3:**
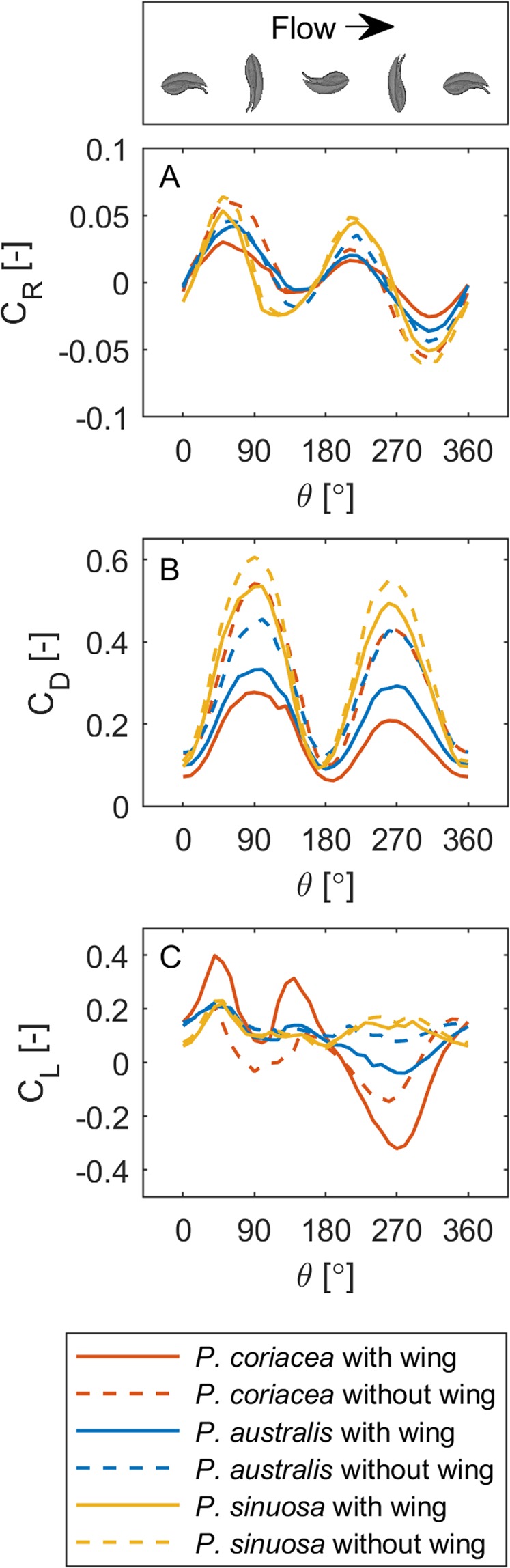
Variation in rotational (**A**), drag (**B**) and lift (**C**) coefficients (normalized by the seed plan area) for *P. coriacea*, *P. australis* and *P. sinuosa* with and without a wing for different positions on the seabed.

A combination of laboratory flume experiments and Computational Fluid Dynamic (CFD) modelling of the 3-dimensional micro-CT scans demonstrated that the large differences in wing width in congeneric species are a direct adaptation to the hydrodynamic environments where each species are found (Table 1). The membranous wing that covers the seed, reduced hydrodynamic rotation, drag and lift forces (Fig. 3). There was a tendency for seeds from all species to rotate in the horizontal plane into a stable position on the bed (Fig. 3A). The greatest stability occurs when the seeds are pointed into the current or when the membranous wing is pointed into the current (Fig. 3A).

Seeds maintain contact with the seabed in the presence of strong turbulence: the larger the wing, the more stable the seed. When all seeds face into the current, drag and lift coefficients (normalised by the seed plan area and represent the respective forces) were small and converged to similar values for all cases (Fig. 3B,C). However, the drag coefficient rapidly increased as the seeds were rotated out of this stable position (Fig. 3B). For *P. coriacea*, the membranous wing could either act to impose lift force on these seeds if they were positioned slightly askew from their most streamlined position, or act to impose a downward force when the seeds were positioned with the membranous wing pointed into the current (Fig. 3C). *Posidonia coriacea* seeds have the most effective wing for reducing hydrodynamic forces that are typical of shallow wave-exposed coastal environment in western and southern Australia (Table 1). The lift coefficients for *P. australis* and *P. sinuosa* were less influenced by the presence of a membranous wing (Fig. 3C), corresponding to their usual habitat, wave sheltered bays and estuaries, where the seeds are able to settle and colonize less energetic environments with reduced forces that could affect their stability (Table 1).

## Discussion

Our results demonstrate for the first time that a unique morphology, a thin membrane covering the seed in the Australian species of the marine genus *Posidonia* has evolved to utilize benthic boundary layer physics to settle and attach to the substratum across environmental gradients in wave and current intensity. This thin seed membrane, provides an essential window of stability until the rapid gravitropic growth of the root^8^ anchors the seed to the bed. An example of convergent evolution among differing taxa, the asymmetric linear shape of this membranous wing and the seeds of seagrasses is similar in form to similar structures utilized by algae^9^ and invertebrate larvae^10^ to settle and attach to the substratum in moving seawater. We hypothesize that this unique seed trait in a marine angiosperm evolved in seagrasses to colonise a wide range of environments to fill available niches from open, high energy coasts to calmer environments in bays and estuaries.

The form of the membranous wing as shown from SEM is quite similar to a winged achene or samara, such as found in maple and ash trees^7^, where papery tissue from the ovary wall develops into a flattened single-sided aerodynamic wing. The wing reduced hydrodynamic rotation, drag and lift forces resulting in the seed being less susceptible to movement on the seabed. Such movement disrupts the gravitropic growth of the root and consequently the success of seedling recruitment at a given location. Seeds of all species rotated in the flow to two weak equilibrium positions parallel to flow direction or where the wing was pointed into the current. Flume experiments with elongated shapes (objects of similar geometrical form to *Posidonia* seeds) in unidirectional flow have demonstrated that the most stable solution for an asymmetrical elongated shape is parallel to flow direction^10^ and that elongated shapes are prone to automatically rotate into a pattern that included two weak equilibrium positions^11^, consistent with our seed rotation results. The wing adds a further dimension, producing a downward force if aligned into the current. Such a downward force has been observed to stabilise flounder on the seabed^12^ and is also deliberately induced through the careful design of wing features on vehicles^13^.

The membranous wing in Australian species of *Posidonia* is a clear demonstration of how form evolves to overcome the physics of settlement and attachment into the benthic boundary layer in the ocean. The need for seeds to maintain a stable position to allow root initiation and growth is similar to the broader issues of settlement and attachment of marine organisms in moving seawater. Some evolved solutions in microbes, algae and invertebrates and other species of seagrass include mucus sheaths in red algae^9^ and marine snails^14^, mucus threads in bacteria^15,16^, larval sea anemones^17^, corals^18,19^ and bivalves^20^, and adhesive hypocotyl hairs in other seagrasses^21,22^. If the seeds are moved by currents, waves or turbulence the strong gravitropic root response is disturbed. This disturbance can result in roots that develop into the water column or become ‘corkscrewed’ as root growth adjusts to being rotated and tumbled at the sediment surface.

Seagrassses are an unusual ancient group of higher flowering plants that have evolved unique traits to establish, grow, reproduce, and survive in the sea (e.g. tolerance to salt water, submarine pollination, reduced cuticle on the leaf surface, no stomata, chloroplasts in the epidermis). While seagrasses are highly clonal, sexual reproduction and their offspring, seeds, are also important in both ecological and evolutionary connectivity^3,4,23,24^. Our results demonstrate, for the first time, that species in the marine genus *Posidonia* have a unique morphology where a thin membrane covering the seed, plays a critical role in its establishment across environmental gradients in wave and current intensity until the seed is able to anchor with rapid growth of the gravitropic root^8^. The primary root is already growing in the direct developing seeds of *Posidonia* and rapidly extends into the sediment by 0.5 to 1 cm within 12–24 hours. This reliance on root growth differs from *P. oceanica* in the Mediterranean where seeds initiate many sticky hypocotyl filaments that attach to rocks and sand grains as a primary root develops^21,22^. The development of membranous wings appears to have evolved after the isolation of Australian congeneric species from *P. oceanica* with the closure of the Tethys Sea during the Miocene (20–10 Myr BP)^25,26^. Sticky hypocotyl filaments like those found in *P. oceanica* have also been described from other seagrass genera including *Zostera*^27^, *Halophila*^28,29^ and *Thalassia*^30^. That hypocotyl filaments are less abundant in Australian *Posidonia* increases the importance of the wing-like adaptation of the membrane covering seeds.

Seagrasses are some of the most threatened habitats in the world’s oceans today^31,32^. Despite providing significant ecosystem services, especially nursery habitat^33^, as well as many ongoing efforts to ameliorate anthropogenic effects that are the leading cause for seagrass decline^34^, attempts to restore seagrass remain elusive^31^. The establishment phase has proved to be a major bottleneck to recovery in many environments^35^. As seagrass seeds are known to be vitally important in the recovery of seagrass meadows^23^, a thorough understanding of how seeds of different seagrass species behave in aqueous environments will be one crucial element in the success of managing seagrass ecosystems for future resilience and in developing successful seed-based restoration strategies similar to those found in terrestrial plant restoration^36^.

## Methods

### Collection of seeds

Approximately 30 fruits, containing an individual seed were collected from inflorescences of *P. coriacea*, *P. australis* and *P. sinuosa*. These collections were made between 2 and 5 m depths using SCUBA at Rottnest Island (S 32.000625°, E 115.548531°), and Parmelia Bank (S 32.096408°, E 115.728296°), Western Australia. The fruit were transferred to large 300–800 L aquaria with recirculating seawater (salinity = 35.6) in a greenhouse until the seeds were released. Seeds were then taken into the laboratory for processing. Maximum wing width was measured from 30 seeds from *P. sinuosa*, *P. australis* and *P. coriacea* with the use of a pair of calipers and a dissecting microscope.

### Scanning electron microscopy (SEM)

Seeds of each species were fixed in a solution of 2.5% glutaraldehyde and 1.7% paraformaldehyde in seawater that was buffered with 0.1 M phosphate buffer. Seeds were stored at 4 °C until SEM. Segments 10–15 mm^2^ in length were excised from different parts of the wings, washed in deionized water, dehydrated in a graded series of ethanol (30–100% and 100%-anhydrous) and then flooded with liquid CO_2_ for 1.5 h before critical point drying (31 °C, 1200 psi). The segments were gold sputter-coated and then examined at 5 kV and 30 µm aperture size with a SEM Zeiss 55.

### X-ray micro-computed Tomography

Each seed was stained with 1% osmium tetroxide (OsO_4_) in 0.1 M phosphate buffer for 6 min using microwaves for better peretration of the stain. Seeds then were mounted in 5% agar in warm water in a 5 ml polypropylene tube. Samples were scanned at 60 kV and 83 µA (5 Watts) using a µCT system (Versa XRM520, Zeiss) running Scout and Scan software (v10.6.2005.12038, Zeiss). A total of 3201 projections were collected over 360°, each with a 2 s exposure. Binning (2x) was used to achieve a suitable signal to noise ratio and 0.4x optical magnification was used to achieve an isotropic voxel resolution of 35.4 µm. No source filters were applied. Projections were reconstructed using XMReconstructor software (v10.7.3679.13921, Zeiss) wih a standard centre shift and beam hardening correction and a 0.7 kernel size reconstruction filter setting. Data generated from µCT scans was analysied and visualized with Avizo (v8.1.1, FEI) software. Workflows for seed segmentation and analysis are provided in Tables S1 and S2. In order to measure only the external surface of the seed and not the internal connecting surfaces of seeds, common (connected) surfaces were removed by subtracting the surface areas measured for relevant combinations of the parts of the seed (as listed in Table S1 step 3). The statistical significance of change (decline) in surface area, volume and surface area to volume ratios for the paired seeds with and without membranous wings were tested using one tailed paired t tests using R.statistics (Version 3.2.4, dplyr package) (Table 2).

### Numerical modelling

Quantifying the forces imposed on seagrass seeds requires high-resolution flow field data at the seed scale. High resolution computational fluid dynamics (CFD) simulations can provide detailed flow field information and also provide direct measurements of canopy element drag forces. Reynolds Averaged Navier Stokes Simulations, RANS-models, split the velocities, *u*, *v*, *w*, and the pressure, *p*, into a mean $\underline{u}$ and fluctuating component $\hat{u}$, i.e. $u=\underline{u}+\hat{u}$. The Navier Stokes equations are averaged both in time and in space and the goal is to capture the average of key processes (e.g., velocities, pressure) in each timestep. RANS models do not include any random turbulent motion, and the turbulent motions are all estimated through sub-scale models and represented by eddy viscosities and diffusivities. For the aim of this study, this approach was considered an appropriate balance of the computational cost and physical process insight.

OpenFoam+ v1712 and the incompressible solver SimpleFoam was used in the analysis with the k-omega-SST turbulence closure model for incompressible flows. A uniform numerical grid was first generated in a 0.2 m × 0.2 m × 0.2 m domain with an initial grid resolution of 5 mm. The seed object obtained from the µCT analysis was then inserted into the domain and the grid further refined. A boundary layer refinement region was defined over the bed and extended to a height of 5 mm above the bed. We then used the SnappyHexMesh algorithm to refine the grid over three layers around seed object. To evaluate the grid-size independence of the numerical solution, results for the default computational grid described above and a finer grid (with resolution increased by approximately 40%) were compared. Differences between numerical results generated with the two grid resolutions were negligible, indicating grid-size-independent solutions.

A mean pressure gradient was imposed in the streamwise direction to drive the flow at the critical velocity determined from laboratory experiments. At the bed and seed surfaces, a no-slip condition was applied. To avoid the complexity of modeling the free surface, the upper boundary of the domain was treated as a frictionless rigid lid. The water depth in all cases was 0.2 m, which ensured a free-stream flow condition developed above the seed. The simulations were allowed to run until a steady state condition was achieved, which was define by monitoring the drag force until this force stabilized. A dynamic adjustable time stepping technique was used to guarantee a local Courant number less than 0.5.

To calculate the rotation, drag and lift forces for each seed case as well as for different seed orientations, the pressure and viscous forces acting on the seeds were calculated. The normalized force coefficients were then determined using established drag, lift and rotation equations (see Supplementary Methods). Simulations and calculations were undertaken at 10 degree resolution, by rotating the seed in the horizontal plane about the seed’s centre of mass.

### Laboratory experiments

The critical velocity to initiate seed movement was quantified for 30 seeds of each species, with and without a membranous wing, in a reticulating flume at the Indian Ocean Marine Research Centre - Watermans Bay (40 cm × 50 cm × 7.5 m). A sediment bed of beach sand (median grain size of 327 µm) was constructed and smoothed in the flume prior to the commencement of each experiment. In each experiment, six seeds were then placed on the bed and the flume slowly filled with seawater (salinity = 35.6) to a depth of 60 cm. The current was slowly increased at 1 cm s^−1^ increments and maintained for 2 min over a current velocity range of 5–48 cm s^−1^. The current velocity was measured at 64 Hz using an acoustic Doppler velocimeter (Nortek ADV) positioned 23 cm above the bed and the velocity acting on the seed determined for the steady two-dimensional flow over a hydrodynamically rough bed using the Karman-Prandtl equation. The position of the seeds were tracked using a downward facing camera at 24 Hz. The video data was transformed into Cartesian coordinates, corrected for distortion and the position of the seeds analysed frame-by-frame for the duration of the experiment. The velocity that initiated seed movement along the bed was defined as the velocity where the seed had the greatest instantaneous movement distance.

## Supplementary information

Supplement Information

Supplement Movie

## Data Availability

3D reconstructions of the seeds from the micro-CT scans are available on the UWA Research Repository (10.26182/5df1a8623d1ea). All other data is available in the main text or the Supplementary Materials.
