# Supplementary material for: A novel adaptation facilitates seed establishment under marine turbulent flows
Source: Sci Rep. 2019 Dec 23;9:19693. doi: 10.1038/s41598-019-56202-7 (PMC6928165; doi:10.1038/s41598-019-56202-7)
Supplement: Supplementary file 1 — Supplement Information [file 41598_2019_56202_MOESM1_ESM.pdf]

Supplementary Materials for

A novel adaptation facilitates seed establishment under marine turbulent flows

Gary A. Kendrick[1,2*], Andrew W.M. Pomeroy[2,3,4],Robert J. Orth[5], Marion L. Cambridge[1,2], Jeremy Shaw[6], Lukasz Kotula[7], Ryan Lowe[2,3]

Affiliations:
[1]The University of Western Australia, School of Biological Sciences.
[2]The University of Western Australia, Oceans Institute.
[3]The University of Western Australia, Oceans Graduate School.
[4]Australian Institute for Marine Science.
[5]Virginia Institute of Marine Science.
[6]The University of Western Australia, Centre for Microscopy Characterisation and Analysis
[7]The University of Western Australia, School of Agriculture and Environment
[*]Correspondence to: gary.kendrick@uwa.edu.au

This PDF file includes:

Supplementary Methods
Supplementary Tables S1, S2
Supplementary Captions for Movie S1

Other Extended Materials for this manuscript include the following:

Movies S1

Supplementary Methods
Rotation, Drag and Lift

A seed will rotate on the seabed when the rotational moment (Q_R) is not in balance ($Q_R \neq 0$). $Q_R$is non-zero when a force applied to the seed at a distance $L$from the seed's centre of rotation is not balanced by an equivalent opposing force. The corresponding coefficient of rotation can then be determined from this moment balance:

$$Q_R = 0.5 C_R \rho_w A_R L u^2 \quad (1)$$

where$C_R$is the is the rotational moment coefficient about the axis specified by the centre of rotation, A_R is a reference area for the coefficient, $\rho_w$is the water density and u is the velocity of the water to which the seed is exposed.

A seed will begin to slip along the bed when the drag force (F_D) exceeds the friction between the bed and the submerged gravitational weight of the seed (F_G) less the lift (F_L) that has developed:

$$F_D = \mu(F_G - F_L) \quad (2)$$

where $\mu$is the static friction coefficient (which was calculated using a friction angle of 30°). The drag acting on an object is defined as:

$$F_D = 0.5 C_D \rho_w A_D u^2 \quad (3)$$

where C_D is the coefficient of drag, A_D is a reference area for the coefficient, $\rho_w$is the water density and u is the velocity of the water to which the seed is exposed.

A seed positioned on the bed and subjected to flow is subjected to a lift force, which develops due to the acceleration of the flow near the seed and results in a vertical pressure gradient develops. This lift force can be both positive (acting to lift an object off the bed) or negative (acting to press an object onto the bed). The lift force can be defined as:

$$F_L = 0.5 C_L \rho_w A_L u^2 \quad (4)$$

where $C_L$is the coefficient of lift and $A_L$is a reference area for the coefficient.

The submerged weight of the seed was calculated as:

$$F_G = mg \quad (5)$$

where m is the seed's submerged mass and $g$is the gravitational acceleration constant.

In our analysis, we calculated the drag forces, lift forces and rotational moments directly in the numerical model. We then calculated the respective coefficients for each case and normalised this coefficient using projected plan area (for A_R, $A_D$and A_L) for each seed species and form (with / without a wing). This enabled comparison of the coefficients for each position

on the bed. The projected plan area was obtained from the numerical model post-processing and verified with the area calculated from from the μCT analysis. For Reynolds numbers above 10^4 (i.e., for cases analyzed in this study), seeds of same species and form are expected to differ only in dimensions but not form, thus we expected that cofficents values of will be approximately constant. This was verified in our analysis by comparing the coefficients for a single velocity applied to all cases as well as the velocity that initiated movement for each individual case based on laboratory experiment results.

.

Table S1. Avizo workflow for segmentation of seagrass seeds

Step	Avizo module	Sub-settings	Description
1.	Extract subvolume		To reduce data cube to minimal size
2.	Non-local means filter	xy planes, search window 21, local neighbourhood 5, similarity value 0.6	To simplify (homogenise) the image intensities to facilitate seagrass seed feature extraction.
3.	Edit new label field	Threshold tool and magic wand tool – applied to all slices	To generate label fields defining the specific regions of interest – including: whole seed, seed kernel, pedicel, major keel, minor keel, whole seed (minus keel, pedicel, seed and minor keel, respectively).
4.	Label analysis	3D, basis measures	To calculate the surface area of each label field.

Table S2. Avizo workflow for calculation of maximum and minimum projected areas – based on filtered data from step 2 in Table S1.

Step	Avizo module	Sub-settings	Description
1.	Mask	Whole seed label from step 3 (Table 1) used as binary image source	To remove background from seed data
2.	Transform editor	Trackball mode	To align the seed to the global axis, with the long axis and major keel of the seed being aligned parallel to the z and y axis, respectively.
3.	Resample transformed image	Lanczos, extended	To re-slice the seed data to the new alignment in step 2.
4.	Image ortho projections	yz, xz, xy, then extract image	To generate projections of the seed profile against the nominated planes and to then extract an image from each projection.
5.	Edit new label field	Threshold tool	To create a label field for the projected image of the seed.
6.	Label analysis	xy planes	To calculate the surface area for each label (seed projection)

Movie S1.

Volume renderings taken from X-ray microCT scans of the seagrass seeds (left) *Posidonia coriacea*, (middle) *Posidonia australis* and (right) *Posidonia sinuosa*.
